# Supplementary material for: Direct Diagnosis of Echovirus 12 Meningitis Using Metagenomic Next Generation Sequencing
Source: Pathogens. 2021 May 17;10(5):610. doi: 10.3390/pathogens10050610 (PMC8156364; doi:10.3390/pathogens10050610)
Supplement: Supplementary file 1 [file pathogens-10-00610-s001.zip › pathogens-1165744-supplementary.pdf]

Supplementary Material 1: Phylogenetic tree based on 3D polymerase encoding gene analysis.

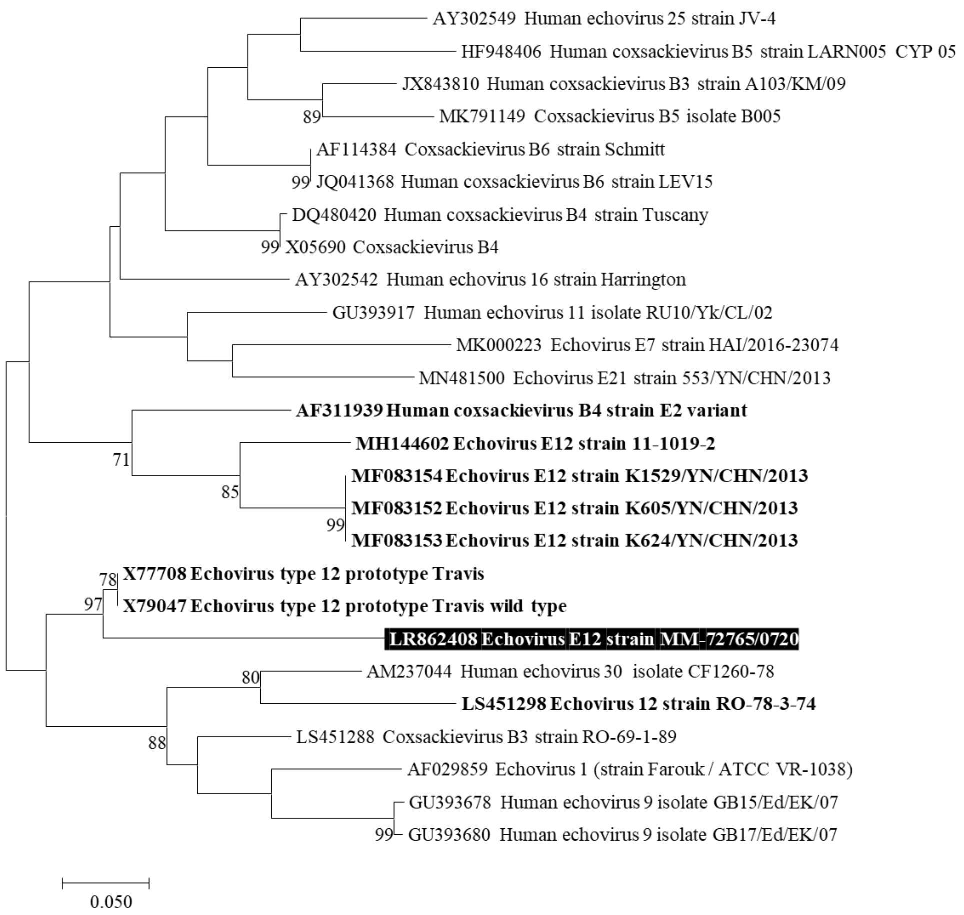

Supplementary Material 1: phylogenetic tree based on *Enterovirus 3DPol gene* encoding sequence**.** The *Echovirus 12* 3Dpol encoding sequence obtained by mNGS (GenBank accession no LR862408; indicated by a white bold font and a black background, and *Echovirus 12* genomes recovered from the NBCI GenBank nucleotide sequence database (http://www.ncbi.nlm.nih.gov/nucleotide/), indicated by a bold font, were incorporated in the phylogeny reconstruction in addition to other enterovirus 3Dpol sequences recovered from GenBank database. The identified 3Dpol gene Echovirus 12 is highly similar to stain prototype Travis, wild type genome (X79047, X77708) and clustered with this this 3Dpol sequence, that confirming the BLAST and VP1 phylogenetic analysis results. The evolutionary history was inferred by using MEGA 7 software version 7.0.2. This analysis involved 26 nucleotide sequences. There was a total of 280 positions in the final dataset. The tree was obtained automatically by applying Neighbor-Join and BioNJ algorithms, Kimura 2-parameter model. The percentage of trees in which the associated taxa clustered together the bootstrap test (1,000 replicates) is shown next to the branches. The tree is drawn to scale, with branch lengths measured in the number of substitutions per site. Bootstrap values >70% are labeled on the tree.

Supplementary Material 2: DNase treatment and cDNA synthesis**.**

DNase treatment

The reaction was performed in thermal cycler 30 minutes at 37 ℃ in 40 µL final volume containing 4 µL of 10x ezDNase Buffer, 4 µL ezDNase enzyme (Thermo Fisher), 12 µL nuclease free water and 20 µL of the extracted RNA. The treated RNA was cleaned by concentrator kit (Zymo Recherch) and eluted in final volume of 20 µL.

cDNA synthesis

For whole genome sequencing, cDNA is synthetized following to the TaqMan (Applied Biosystem) protocol, in a final volume of 50 µL containing 50 µM MgCl2, 10x buffer, 10 mM dNTPs, 1 µM random primers, 5 units of Superscript III and 5 µL RNase inhibitor, then the volume was adjusted to 50 µL with the extracted RNA. cDNA synthesis reaction was performed in Thermal cycler (Applied Biosystem) following a standard protocol, 10 minutes at 25 ℃, 30 minutes at 48 ℃ and 5 minutes at 95 ℃ to inactivate the RNase inhibitor. The doubles strand cDNA was performed in 30 µL-volume containing 10x Neb buffer 2, 10 µM dNTPs, nuclease free water, 3 unites of DNA Polymerase I, Large (Klenow) Fragment (BioLabs) and 20 µL cDNA. The double strand synthesis takes 2 hours at 37 ℃, then was purified using 0.5 Ratio Agencourt® AMPure beads and eluted in 20 µL-volume of 1x-sterile Tris-EDTA solution.

Supplementary Material 3: NGS Library preparation.

The NGS library sequencing was performed following the manufacturer Illumina iSeq library preparation protocol. Briefly, 5 µL of standardized DNA were fragmented in a mix containing 5 µl of Amplicon Tagment Mix in presence of Tagment DNA Buffer (Nextera XT Library prep Kit, Illumina) for 5 min at 55 ℃, in a ABI 2720 GeneAmp PCR System Thermal Cycler (Applied Biosystems, Foster City, CA, USA) in a 20 µL volume. Then 5 µL of Neutralize Tagment Buffer were added before centrifugation for 1 min at 2,800g and 5-min incubation at room temperature, then amplified in a 50 µL volume, in presence of Nextera ® XT Index Kit V2, (Nextera ®, San Diego, USA). A first purification was performed using Agencourt Ampure XP beads (Beckman Coulter, Villepinte, France) in a 0.8 ratio of beads followed by two washes with 80% alcohol, then elution in 52.5 µL of RSB buffer. The library concentration measured in Agilent 2100 Bioanalyzer (Thermo Fisher Scientific), then diluted in RSB buffer in presence of 10 µL-volume of Phix (50 pM). Finally, 50 pM of the diluted libraries was denatured and sequenced on the iSeq 100 sequencer (Illumina) in a single 17.5-h run providing 2x150-bp long reads.

Supplementary Material 4:

Primer F	5’-AGTGCAGGGTACCCGTATGT-3‘
Primer R	5’-GGTCCGGATCACATCCAACT-3’

>AGTGCAGGGTACCCGTATGTCGCCATAGGCATCAAAAAGAGAGACATCCTATCCAAAAAGACTA
AAGATCTGACTAAATTGAAGGAATGCATGGACAAGTACGGATACCCGTAGGTCAGTCACTGATTAC
GGTTTCCTGAGACCTCCCACACACAGTTCTCCGAGTCAATCATGACTGGCTAGGTGTCTAGTTTAAA
TGACTCTGTCGCGATGAGCCAGGCAACTTGTACAAAACATTCCATCTGAACCCGGGCATTGTTACA
GGCAGTGCAGTTGGATGTGATCCGGACC.
